# The utility of the rapid emergency medicine score (REMS) compared with SIRS, qSOFA and NEWS for Predicting in-hospital Mortality among Patients with suspicion of Sepsis in an emergency department

**DOI:** 10.1186/s12873-020-00396-x

**Published:** 2021-01-07

**Authors:** Onlak Ruangsomboon, Phetsinee Boonmee, Chok Limsuwat, Tipa Chakorn, Apichaya Monsomboon

**Affiliations:** Department of Emergency Medicine, Faculty of Medicine, Siriraj Hospital, Mahidol University, 2 Wanglang Road, Bangkoknoi, Bangkok, 10700 Thailand

**Keywords:** Sepsis, Early warning score, Rapid emergency medicine score

## Abstract

**Background:**

Many early warning scores (EWSs) have been validated to prognosticate adverse outcomes secondary to sepsis in the Emergency Department (ED). These EWSs include the Systemic Inflammatory Response Syndrome criteria (SIRS), the quick Sequential Organ Failure Assessment (qSOFA) and the National Early Warning Score (NEWS). However, the Rapid Emergency Medicine Score (REMS) has never been validated for this purpose. We aimed to assess and compare the prognostic utility of REMS with that of SIRS, qSOFA and NEWS for predicting mortality in patients with suspicion of sepsis in the ED.

**Methods:**

We conducted a retrospective study at the ED of Siriraj Hospital Mahidol University, Thailand. Adult patients suspected of having sepsis in the ED between August 2018 and July 2019 were included. Their EWSs were calculated. The primary outcome was all-cause in-hospital mortality. The secondary outcome was 7-day mortality.

**Results:**

A total of 1622 patients were included in the study; 457 (28.2%) died at hospital discharge. REMS yielded the highest discrimination capacity for in-hospital mortality (the area under the receiver operator characteristics curves (AUROC) 0.62 (95% confidence interval (CI) 0.59, 0.65)), which was significantly higher than qSOFA (AUROC 0.58 (95%CI 0.55, 0.60); *p* = 0.005) and SIRS (AUROC 0.52 (95%CI 0.49, 0.55); *p* < 0.001) but not significantly superior to NEWS (AUROC 0.61 (95%CI 0.58, 0.64); *p* = 0.27). REMS was the best EWS in terms of calibration and association with the outcome. It could also provide the highest net benefit from the decision curve analysis. Comparison of EWSs plus baseline risk model showed similar results. REMS also performed better than other EWSs for 7-day mortality.

**Conclusion:**

REMS was an early warning score with higher accuracy than sepsis-related scores (qSOFA and SIRS), similar to NEWS, and had the highest utility in terms of net benefit compared to SIRS, qSOFA and NEWS in predicting in-hospital mortality in patients presenting to the ED with suspected sepsis.

**Supplementary Information:**

The online version contains supplementary material available at 10.1186/s12873-020-00396-x.

## Introduction

Sepsis is a state of organ dysfunction caused by dysregulated host response to infection [1, 2]. It is a critical condition with a high mortality rate and is considered a major health problem worldwide [1–3]. In high-income countries, around 20 million people suffer from sepsis each year with mortality rate ranging between 17 and 26% [4]. In the middle-income country of Thailand, the mortality rate was estimated at 25–50% [5, 6]. Thailand has limited universal coverage health care resources with increasing emergency department (ED) overcrowding. Thus, the burden of disease is higher than in high-income countries.

Early recognition of patients with sepsis is the key to improve its management, especially in those with greater severity who are at risk of adverse outcomes. Developing early warning score (EWS) tools to identify these patients early may aid clinicians to accelerate treatment and could lead to improved outcomes. Sepsis was previously defined and identified using Systemic Inflammatory Response Syndrome criteria (SIRS). However, SIRS has been criticized for its low specificity [7–9], which led to the introduction of the quick Sequential Organ Failure Assessment (qSOFA) proposed by the third international consensus definition (Sepsis-3) [10]. qSOFA has been shown to have better specificity but lower sensitivity than SIRS. Consequently, it might not detect patients early enough in their course of disease and may not be beneficial for ED utilization [11–14].

Many EWSs have been developed for ED use. The National Early Warning Score (NEWS) was developed to assess and monitor hospitalized patients for early detection of clinical deterioration [15]. Despite being developed for clinical deterioration, it has been validated as a feasible predictor for adverse outcomes due to sepsis. NEWS has higher accuracy than qSOFA and SIRS for predicting mortality and intensive care unit (ICU) transfer of suspected septic patients [16–19]. The Rapid Emergency Medicine Score (REMS) was developed to predict in-hospital mortality in non-surgical ED patients [20]. It has not been validated and compared to sepsis-related scoring systems and other EWSs to predict adverse outcomes due to sepsis. Thus, we aimed to validate and compare the clinical utility of REMS, SIRS, qSOFA, and NEWS in predicting in-hospital mortality and mortality within 7 days of admission in ED patients with suspected sepsis.

## Methods

### Study design and setting

We conducted a retrospective study at the ED of Siriraj Hospital, Mahidol University, Bangkok, Thailand. Siriraj Hospital is the largest tertiary university hospital in Thailand with over 20,000 Emergency Severity Index level 1–2 ED visits per year. Siriraj Institutional Review Board approved the study. Patients’ inform consent was waived due to de-identification of their data.

### Patients

Adult patients aged > 18 years were eligible if they were suspected of having sepsis by ED physicians using clinical judgement and had hemoculture taken, followed by prescribed intravenous antibiotics or vice versa. Patients transferred from outpatient units after having been treated with intravenous antibiotics were excluded.

### Data collection

We assessed ED patients retrospectively for eligibility between 1 August 2018 and 31 July 2019. Triage nurses assessed patients visiting the ED and recorded their initial vital signs in the standing triage form, and then attending ED physicians assessed them.

We extracted physiologic variables, underlying conditions, management, and outcomes from electronic medical records. All components of each risk score were in the standing ED admission triage form, from which we used the initial values at presentation to retrospectively calculate all risk score values. If sepsis was suspected after 4 h from time of ED visit, we imputed the values closest to the time of suspicion, defined as time of culture or antibiotics, whichever came first. We calculated all risk scores using online calculator (MdCalc online calculator).

### Scoring systems

SIRS is a 4-item score consisting of pulse rate, respiratory rate, body temperature and white blood cell counts; each item containing 1 point (0–4 points). qSOFA has 3 items with 1 point each; respiratory rate, mental status and systolic blood pressure (0–3 points). NEWS and REMS are scoring systems with multiple components with weighted score points. NEWS (0–20 points) consists of pulse rate, respiratory rate, body temperature, systolic blood pressure, oxygen saturation and need for oxygen supplement. REMS comprises of pulse rate, respiratory rate, mean arterial pressure, mental status, pulse oximetry and age (0–26 points). The components and details of each risk score are presented in the Table S1.

### Outcomes

The primary outcome was all-cause in-hospital mortality, and the secondary outcome was all-cause mortality within 7 days of ED visit.

### Statistical analysis

We reported categorical variables as frequency (percentage) and continuous variables as mean (SD) or median (interquartile range) as appropriate. We compared patient characteristic variables using Chi square, t-test, or Mann-Whitney U test as appropriate.

The predictive performance of SIRS, qSOFA, NEWS, and REMS for primary and second outcomes was assessed. Discrimination was assessed by area under the curve of the receiver operator characteristics curves (AUROC). We estimated the 95% confidence interval (CI) of the AUROCs and made comparisons between EWSs using a bootstrapped method at 10000 replications. We evaluated calibration with calibration plots and the Hosmer-Lemeshow test, using a smoothed nonparametric method to fit the calibration curves [21, 22]. Overall model performance was tested by scaled Brier score and Nagelkerke’s R squared. A complementary analysis of EWSs was performed incorporating information an ED physician may have at assessment including age, gender, and Charlson Comorbidity Index. Baseline mortality risk models were fitted for each outcome with age as a restricted cubic spline [21]. The additional predictive contribution of each EWS to the baseline risk model was assessed by likelihood ratio test. Comparisons between baseline risk model plus an EWS versus baseline risk model plus a different EWS were assessed by bootstrap test with 10,000 replications. Integrated discrimination improvement assessed whether a baseline plus EWS model had better discrimination than the baseline model alone by difference in discrimination slopes between the models [23].

Good discrimination and calibration may not reflect the clinical usefulness of an EWS because they assign equal weight to sensitivity, specificity, and prediction errors. ED physicians making decisions in clinical practice usually assign different weights to these based on the patient’s characteristics and available resources. To reflect this, we calculated net benefit (NB) at each threshold probability from decision curve analysis [24]. For a patient with suspected sepsis, the ED physician weights the harm/cost of overtreatment against the benefit of treatment (the harm/cost-to-benefit ratio). If the physician thinks that the harm/cost-to-benefit ratio is 1:9, this represents a threshold probability of 10%, and the number of patient that the physician is willing to treat (NWT) to prevent the mortality outcome is 10. We present a threshold probability range from 1 to 20% (NWT from 100 to 5, respectively), which is a plausible range over which a physician would use an EWS for a patient with suspected sepsis. A higher NB is desirable at any threshold probability and should be higher than ‘treat all’ or ‘treat none’ strategies to have clinical utility.

Clinical usefulness at cutoff values was also assessed by sensitivity, specificity, positive likelihood ratio (LR+), negative likelihood ration (LR-), negative predictive value (NPV) and positive predictive value (PPV). These were calculated for SIRS and qSOFA at the recommended cutoffs from previous literature [10, 25]. For NEWS and REMS, we reported the accuracy at the optimal cut-point according to optimal Youden index. Pre-specified subgroup analyses were performed by age ≥ 70 years and age < 70 years as well as by no comorbidities and at least one comorbidity. Comorbidities included chronic neurologic disease, acute stroke, chronic respiratory disease, chronic liver disease, neoplasia, chronic renal disease, diabetes mellitus, chronic heart failure, and immunocompromised status.

All statistical analyses were performed using R software version 3.6.1 (R Foundation for Statistical Computing, Vienna, Austria) with the rms, Hmisc, foreign, pROC, sciplot, and dca packages except for sensitivity and specificity, LR+, LR-, NPV and PPV, which were calculated using MedCalc for Windows version 19 (MedCalc statistical software, Mariakerke, Belgium).

## Results

### Study population

A total of 15,830 patients visited the ED during August 2018–July 2019. Of these, 1927 (12.2%) patients had suspected sepsis, and 305 were excluded because they had been treated and transferred from other units. Consequently, 1622 patients with suspected sepsis were included in the final analysis. Of these, 457 (28.2%) met the primary outcome of all-cause in-hospital mortality, and 280 (17.3%) died within 7 days of admission. A total of 1382 (85.2%) were diagnosed with sepsis at hospital disposition according to Sepsis-3 definition. Patient characteristics are shown in Table 1. The study population’s mean age ± SD was 72.6 ± 15.4 years, and 51.1% were female. Patients who had all-cause in-hospital mortality were older, had a greater prevalence of neoplasia, and more history of recent hospital admission. They also had significantly more severe abnormal initial vital signs, higher serum white blood cells and band-form cell counts, as well as higher rate of positive hemoculture, inotropic drug prescription and ICU admission compared with patients discharged alive.

**Table 1:** Baseline characteristics of patients with suspected sepsis

Characteristic	All (1622)	Dead (457)	Alive (1165)	***p***-value
Age	72.6 ± 15.4	74.4 ± 15.1	71.9 ± 15.5	0.004
Sex (female)	829 (51.1)	233 (51.0)	596 (51.2)	0.95
Underlying disease				
Diabetes mellitus	513 (31.6)	144 (31.5)	369 (31.7)	0.50
Hypertension	888 (54.7)	236 (51.6)	652 (56.0)	0.07
Hyperlipidaemia	525 (32.4)	160 (35.0)	365 (31.3)	0.09
CKD or ESRD	294 (18.1)	82 (17.9)	212 (18.2)	0.48
Coronary artery disease	206 (12.7)	51 (11.2)	155 (13.3)	0.14
Neuro-debilitating diseases	401 (24.7)	111 (24.2)	290 (24.8)	0.40
Cancer	404 (24.9)	164 (35.9)	240 (20.6)	< 0.001
Recent admission< 3 months	729 (44.9)	245 (53.6)	484 (41.5)	< 0.001
Infection site				
Respiratory tract	982 (60.5)	294 (64.3)	688 (59.1)	0.04
Urinary tract	198 (12.2)	44 (9.6)	154 (13.2)	
Other known sites	139 (8.6)	26 (5.7)	113 (9.7)	
Unknown	303 (18.7)	93 (20.4)	210 (18.0)	
Type of infection				
Community-acquired	830 (51.2)	202 (44.2)	628 (53.9)	< 0.001
Healthcare-associated	89 (5.5)	18 (3.9)	71 (6.1)	
Hospital-associated	703 (43.3)	237 (51.9)	466 (40.0)	
Vital signs and mental status at time of sepsis suspicion				
Body temperature (°C)	37.8 ± 8.4	38.1 + 3.7	37.6 + 3.7	0.40
Respiratory rate (breaths/min)	31.1 ± 8.6	32 + 8.1	30.8 + 8.7	0.01
Pulse rate (beats/min)	102.6 ± 38.4	102.9 + 27.4	102.5 + 42	0.80
Systolic blood pressure (mmHg)	125.4 ± 37.3	121 + 43.5	127.6 + 34.3	0.01
Diastolic blood pressure (mmHg)	71.3 ± 26.7	69.1 + 19.3	72.1 + 19.4	0.016
Mean arterial pressure (mmHg)	89.3 ± 26.2	86.4 + 23.8	90.4 + 26.9	0.004
Oxygen saturation (%)	91.9 ± 8.7	89.9 + 10.2	92.7 + 7.8	< 0.001
Glasgow coma scale score	12.5 ± 2.5	11.2 ± 2.9	13.0 ± 2.0	< 0.001
Early warning scores at time of sepsis suspicion				
SIRS	2.40 ± 0.97	2.47 ± 0.90	2.37 ± 0.99	0.08
qSOFA	1.38 ± 0.65	1.52 ± 0.70	1.32 ± 0.62	< 0.0001
NEWS	8.05 ± 3.30	8.90 ± 3.37	7.71 ± 3.21	< 0.0001
REMS	9.08 ± 3.12	10.06 ± 3.12	8.70 ± 3.00	< 0.0001
Laboratory results				
White blood cells count (cells/mm^3^)	12,822.5 ± 9514.8	13,474.1 ± 9755.8	12,566.9 ± 9410.5	0.08
Band form (%)	2.5 ± 1.0	3.9 ± 1.6	1.9 ± 0.6	< 0.001
Hemoculture positive	276 (17.0)	100 (21.9)	176 (15.1)	0.001
ED management				
Inotropic drugs	340 (21.0)	153 (33.5)	187 (16.1)	< 0.001
Outcome				
Length of hospital stay (days)	6 (3,12)	5 (2,12)	7 (3,12)	0.38
ED disposition				
ICU admission	82 (51.0)	38 (8.3)	44 (3.8)	< 0.001

Data presented as n (%), mean ± SD or median (IQR)

*Abbreviations: CKD* chronic kidney disease, *ESRD* end-stage renal disease, *SIRS* systemic inflammatory response syndrome, *qSOFA* quick Sequential Organ Failure Assessment, *NEWS* National Early Warning Score, *REMS* Rapid Emergency Medicine Score, *ICU* intensive care unit

### Scoring systems

**Fig. 1:**
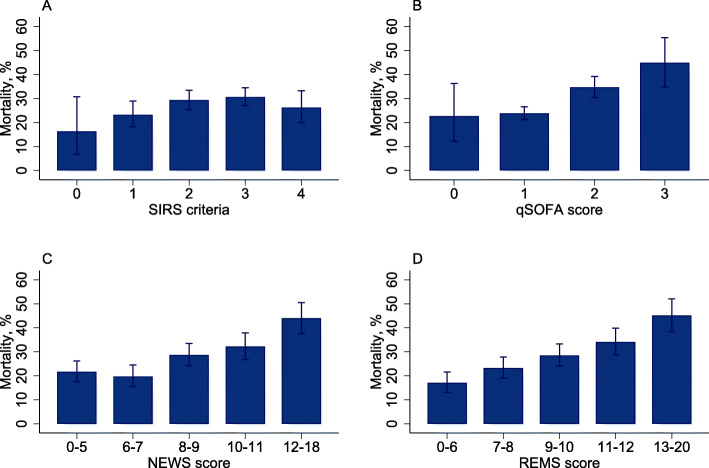
In-hospital mortality stratified by each early warning score in patients with suspected sepsis. **a** SIRS criteria. **b** qSOFA score. **c** NEWS score. **d** REMS score. Error bars denote 95% confidence intervals. NEWS and REMS scores were categorized into quintiles of score. Abbreviations: NEWS, National Early Warning Score; qSOFA, quick Sequential Organ Failure Assessment; REMS, Rapid Emergency Medicine Score; SIRS, systemic inflammatory response syndrome

**Fig. 2:**
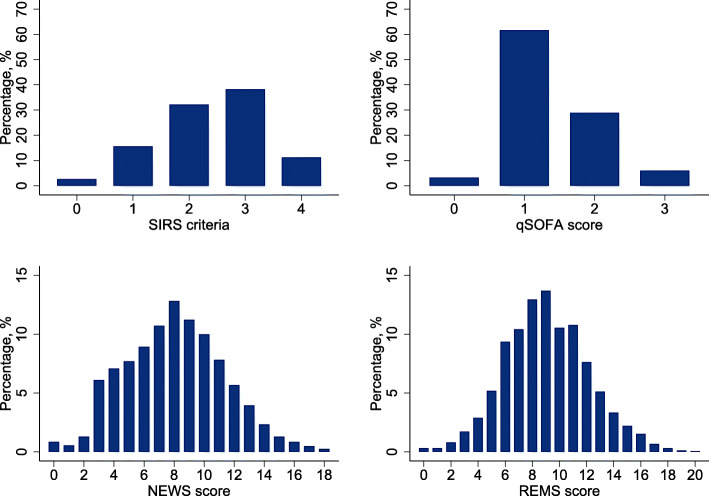
The distribution of early warning score in patients with suspected sepsis. **a** SIRS criteria. **b** qSOFA score. **c** NEWS score. **d** REMS score. Abbreviations: SIRS, systemic inflammatory response syndrome; qSOFA, quick Sequential Organ Failure Assessment; NEWS, National Early Warning Score; REMS, Rapid Emergency Medicine Score

None of the 1622 patients with suspected sepsis had missing EWS values. All 4 mean score values except SIRS were significantly higher in those who died (Table 1). qSOFA and REMS showed clear association with both mortality outcomes whereas higher SIRS did not, and all-cause in-hospital mortality rate did not increase between NEWS 0–5 and 6–7 (Fig. 1 and Fig. S1). Distributions of scores amongst the cohort are shown in Fig. 2.

### Score performance

Overall performance assessed by scaled Brier score and Nagelkerke’s R square showed REMS had the best overall performance, followed by NEWS, qSOFA, and SIRS (Table 2).

**Table 2:** Early warning score performance and clinical utility for all-cause in-hospital mortality and mortality within 7 days of admission in patients with suspected sepsis

	Discrimination	Calibration	Overall performance		Clinical utility						
Score	AUROC (95%CI)	Hosmer-Lemeshow Test	Scaled Brier Score (%)	Nagelkerke’s R-Square (%)	Score category	Sensitivity (95%CI)	Specificity (95%CI)	PPV (95%CI)	NPV (95%CI)	LR+ (95%CI)	LR- (95%CI)
**In-hospital mortality**											
SIRS	0.524 (0.494, 0.553)	0.074	0.2	0.2	SIRS ≥ 2	85.6 (82–88.7)	19.8 (17.6–22.2)	29.5 (28.5–30.5)	77.8 (73.1–81.8)	1.1 (1.0–1.7)	0.7 (0.6–0.9)
qSOFA	0.577 (0.549, 0.604)	0.688	2.0	1.9	qSOFA ≥ 2	45.3 (40.7–50)	69.0 (66.3–71.7)	36.4 (33.4–39.6)	76.3 (74.6–77.9)	1.5 (1.3–1.7)	0.8 (0.7–0.9)
NEWS	0.606 (0.575, 0.636)	0.059	2.7	2.6	NEWS ≥ 8	68.1 (63.7–72.3)	47.8 (44.9–50.7)	33.8 (32.0–35.7)	79.2 (76.7–81.5)	1.3 (1.2–1.4)	0.7 (0.6–0.8)
REMS	0.623 (0.593, 0.653)	0.565	4.0	3.8	REMS ≥ 9	66.5 (62–70.8)	50.1 (47.2–53)	34.4 (32.4–36.3)	79.2 (76.8–81.5)	1.3 (1.2–1.5)	0.7 (0.6–0.8)
**Mortality within 7 days of admission**											
SIRS	0.539 (0.504, 0.571)	0.025	0.3	0.3	SIRS ≥ 2	88.6 (84.3–92.1)	19.8 (17.7, 22.0)	18.7 (18.0–19.5)	89.2 (85.5–92.1)	1.1 (1.1–1.2)	0.6 (0.4–0.8)
qSOFA	0.589 (0.556, 0.622)	0.154	1.6	1.6	qSOFA ≥ 2	49.3 (43.3–55.3)	68.5 (65.9–71.0)	24.6 (22.0–27.3)	86.6 (85.2–88.0)	1.6 (1.4–1.8)	0.7 (0.7–0.8)
NEWS	0.625 (0.589, 0.660)	0.189	2.7	2.7	NEWS ≥ 8	70.0 (64.3–75.3)	46.1 (43.4–48.8)	21.3 (19.8–22.9)	88.0 (85.9–89.9)	1.3 (1.2–1.4)	0.7 (0.5–0.8)
REMS	0.645 (0.608, 0.679)	0.234	4.0	3.8	REMS ≥ 10	57.5 (51.5–63.4)	60.8 (58.1–63.4)	23.4 (21.3–25.7)	87.3 (85.6–88.8)	1.5 (1.3–1.7)	0.7 (0.6–0.8)

Cut-off values for SIRS and qSOFA were chosen from the literature. Cut-off values for NEWS and REMS were chosen by optimal Youden Index

*Abbreviations: AUROC* area under the receiver operator characteristics curve, *CI* confidence interval, *LR+* positive likelihood ratio, *LR-* negative likelihood ratio, *NEWS* National Early Warning Score, *NPV* negative predictive value, *PPV* positive predictive value, *qSOFA* quick Sequential Organ Failure Assessment, *REMS* Rapid Emergency Medicine Score, *SIRS* systemic inflammatory response syndrome

**Fig. 3:**
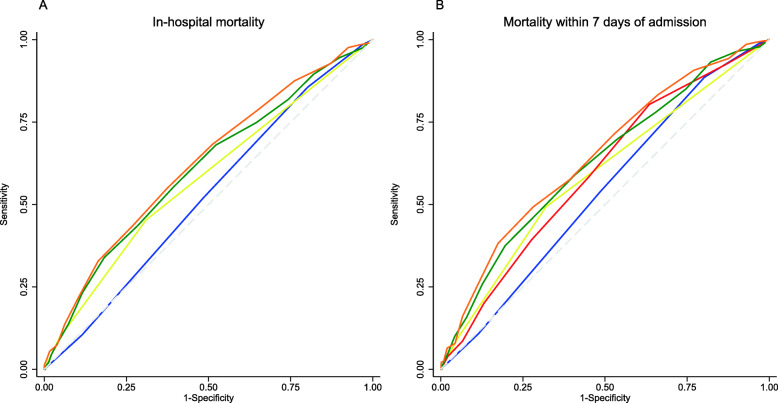
Receiver operator characteristic curves for early warning scores for in-hospital mortality and mortality within 7 days in patients with suspected sepsis. **a** In-hospital mortality. **b** Mortality within 7 days of admission. EWS score = SIRS (blue line), qSOFA (yellow line), NEWS (green line), and REMS (orange line). Abbreviations: SIRS, systemic inflammatory response syndrome; qSOFA, quick Sequential Organ Failure Assessment; NEWS, National Early Warning Score; REMS, Rapid Emergency Medicine Score

**Fig. 4:**
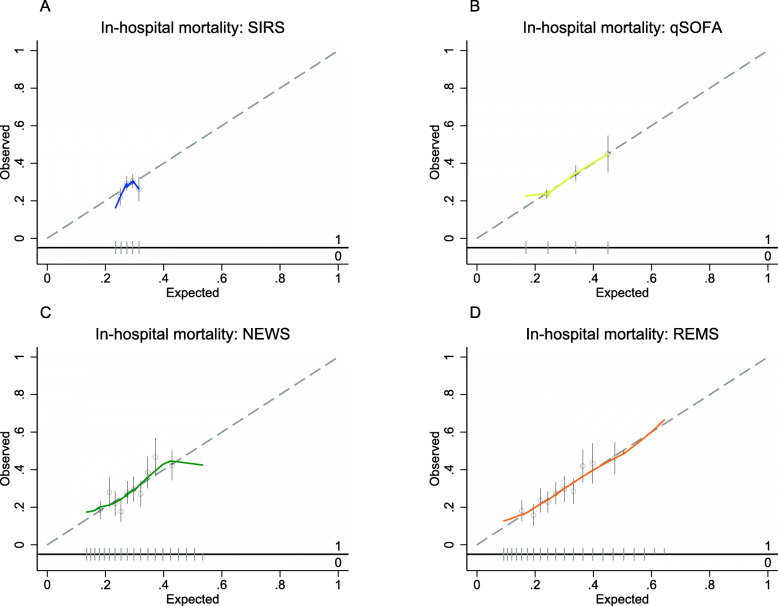
Calibration plots of early warning scores for all-cause in-hospital mortality in patients with suspected sepsis. **a** SIRS criteria. **b** qSOFA score. **c** NEWS score. **d** REMS score. Hollow circles denote groups of predicted risk. Vertical line through hollow circles denote 95% confidence intervals. The distribution of non-events of the outcome (0) and events of the outcome (1) by expected probability are denoted by the rug plot (light grey) along the x axis. Abbreviations: SIRS, systemic inflammatory response syndrome; qSOFA, quick Sequential Organ Failure Assessment; NEWS, National Early Warning Score; REMS, Rapid Emergency Medicine Score

The discrimination performance for all-cause in-hospital mortality was highest for REMS (AUROC 0.62; 95%CI 0.59, 0.65), followed by NEWS (AUROC 0.61; 95%CI 0.58–0.64), qSOFA (AUROC 0.58; 95%CI 0.55–0.61), and SIRS (AUROC 0.52; 95%CI 0.49–0.55) (Table 2 and Fig. 3). All EWSs had better discrimination by AUROCs for all-cause mortality within 7 days of admission compared to all-cause in-hospital mortality although the trend of results of AUROCs was similar (Table 2 and Fig. 3). In pairwise comparisons between EWSs, REMS had significantly better discrimination than all other EWSs except for NEWS for both outcomes (Table 3). In subgroup analyses, all EWSs show better discrimination for all-cause in-hospital mortality and mortality within 7 days in those aged greater or equal to 70 years than those aged less than 70 years (Table S2). All EWSs show better discrimination for all-cause mortality in those without chronic comorbidities compared with those with at least one chronic comorbidity, but an opposite trend was seen for all-cause mortality within 7 days of admission for SIRS and NEWS (Table S2). Calibration for SIRS showed underestimation of predicted mortality risk at lowest and highest SIRS scores (Fig. 4 and S2). The other EWSs tended to be well-calibrated except for at underestimation of all-cause in-hospital mortality risk at high predicted probabilities in NEWS and for all-cause mortality within 7 days of admission for both NEWS and REMS (Fig. 4 and S2). However, only a few patients had very high NEWS and REMS scores (Fig. 2).

**Table 3:** Pairwise comparisons of area under the receiver operator characteristic curve of early warning scores for in-hospital mortality and mortality within 7 days among patients with suspected sepsis

		In-hospital mortality			
		SIRS	qSOFA	NEWS	REMS
**Mortality within 7 days**	**SIRS**		**0.007	*** < 0.001	*** < 0.001
	**qSOFA**	*0.03		*0.05	**0.005
	**NEWS**	*** < 0.001	*0.04		0.27
	**REMS**	*** < 0.001	**0.004	0.26	

Comparison were performed by bootstrap test. *p < 0.05 **p < 0.01 ***p < 0.001

*Abbreviations: NEWS* National Early Warning Score, *qSOFA* quick Sequential Organ Failure Assessment, *REMS* Rapid Emergency Medicine Score, *SIRS* systemic inflammatory response syndrome

### Additional contribution of EWSs to baseline mortality risk model

All baseline risk model plus an EWS had significantly better discrimination than the baseline risk model for all-cause in-hospital mortality (Table S3). For all-cause in-hospital mortality, the baseline risk model plus REMS showed the greatest improvement in discrimination over the baseline mortality risk models, followed by NEWS, qSOFA, and SIRS (Table S4 and Fig. S3). NEWS and REMS had significantly better discrimination than SIRS and qSOFA, but REMS was not significantly superior to NEWS (Table S3). The trend of results was generally similar for all-cause mortality within 7 days of admission except that NEWS did not have significantly better discrimination than qSOFA for both mortality outcomes (Table S3 and S4 and Fig. S3). Integrated discrimination improvement also showed REMS had the greatest improvement over the baseline risk models, followed by NEWS, qSOFA, and SIRS (Table S4). REMS showed the greatest percentage improvement in sensitivity for all-cause in-hospital mortality and all-cause mortality within 7 days of admission compared with the baseline risk model (Table S4). Calibration plots for baseline risk model plus EWSs for both mortality outcomes generally were well-calibrated up to a predicted probability of 0.5 except for SIRS for all-cause in-hospital mortality. Above a predicted probability of 0.5 some models showed some over- or under-estimation of mortality risk (Figs. S4 and S5).

The clinical usefulness of the EWS scores was assessed by sensitivity, specificity, PPV, NPV, LR+, and LR- (Table 2). For all-cause in-hospital mortality, SIR > 2 had the highest sensitivity but the least specificity. qSOFA > 2 had the highest specificity but lowest sensitivity. At optimal Youden Index cut points, NEWS > 8 and REMS > 9 had a balance of sensitivity and specificity, which favored sensitivity. PPV and NPV were similar for all EWSs except for a much lower PPV of SIRS. qSOFA had the highest LR+ while LR- of all EWSs were similar. For all-cause mortality within 7 days of admission, results of sensitivity and specificity were similar except that the optimal cut-off point for REMS was > 10, and results for PPV, NPV, LR+, and LR- were similar to the primary outcome.

In subgroup analysis for all-cause in-hospital mortality, results were generally similar to the full cohort except that REMS > 9 had higher specificity in those aged less than 70 years and higher sensitivity in those aged greater or equal to 70 years (Table S5). Subgroup analysis of all-cause mortality within 7 days of admission showed similar changes to all-cause in-hospital mortality in sensitivity and specificity for REM > 10 (Table S5).

**Fig. 5:**
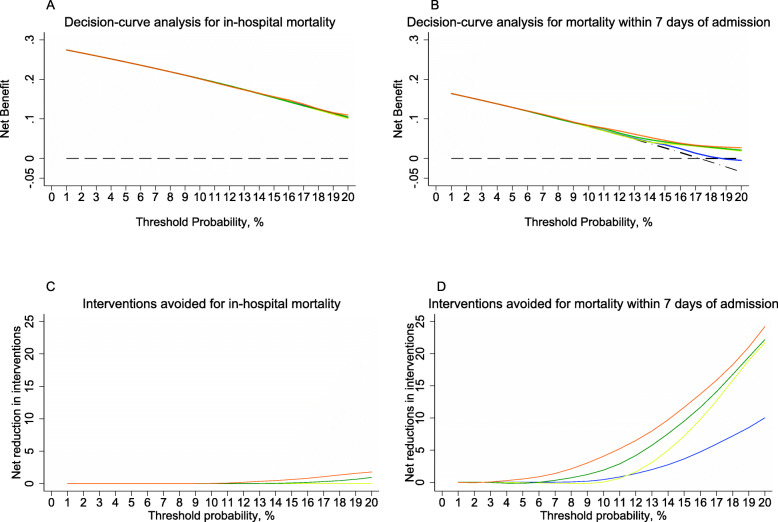
Decision curves comparing the net benefit of SIRS criteria, qSOFA, NEWS and REMS for patients with suspected sepsis at risk of (**a**) in-hospital mortality and (**b**) mortality within 7 days of admission over a plausible range of threshold probabilities. SIRS criteria (blue line), qSOFA (yellow line), NEWS (green line), and REMS (orange line), a ‘treat all’ strategy (black dashed and dotted line), and a ‘treat none’ strategy (black dashed line). Net benefit = (true-positive classifications – harm/cost-to-benefit ratio × false-positive classifications) ÷ N. The threshold probability represents the number of patients that the physician is willing to treat. Net reduction in interventions per 100 patients in a theoretical population for (**c**) in-hospital mortality and (**d**) mortality within 7 days of admission. The distribution of non-events of the outcome (0) and events of the outcome (1) by expected probability are denoted by the rug plot (light grey) along the x axis. Abbreviations: NEWS, National Early Warning Score; qSOFA, quick Sequential Organ Failure Assessment; REMS, Rapid Emergency Medicine Score; SIRS, systemic inflammatory response syndrome

The NB for all-cause in-hospital mortality showed SIRS and qSOFA did not have an advantage over a treat-all strategy for all plausible threshold probabilities. The range of threshold probabilities over which any NB advantage over a treat-all strategy was 18–20% for NEWS and 14–20% for REMS. The number of avoided interventions per 100 patients at a threshold probability of 20% (NWT 5) using NEWS or REMS would be 1.1 and 2.6, respectively (Fig. 5). For all-cause mortality within 7 days of admission, all EWSs showed advantageous NB over a treat-all strategy within the plausible threshold probability range. NEWS and REMS had the lowest threshold probabilities at which advantage over a treat-all strategy began at 10% (NWT 10) and 4% (NWT 25), respectively. The number of avoided interventions per 100 patients in a hypothetical population at threshold probabilities of 10 and 20% for NEWS would be 2.4 and 23, respectively, and for REMS would be 2.4 and 25, respectively (Fig. 5). Results of NB analysis for baseline risk model + EWS were similar for both mortality outcomes (Fig. S6).

Eighty-two percent (*n* = 1325) of all patients with suspected sepsis met at least 2 SIRS criteria (SIR > 2), and 70% (*n* = 934) did not meet the primary outcome (false positive). Only 35% (*n* = 568) met at least 2 qSOFA criteria (qSOFA ≥ 2), and about 64% (*n* = 361) were false positive. NEWS ≥ 8 and REMS ≥ 9 could detect similar proportions of patients (about 56%), but REMS ≥ 9 had the highest absolute risk difference compared to the other three EWSs. Similarly, REMS  10 could also provide the highest absolute risk difference in predicting mortality within 7 days of admission (Table 4).

**Table 4:** Classification according to sepsis criteria

Outcomes	All patients, no (%)	SIRS, no (%)		Absolute difference, % (95%CI)	qSOFA, no (%)		Absolute difference, % (95%CI)	NEWS, no (%)		Absolute difference, % (95%CI)	REMS, no (%)		Absolute difference, % (95%CI)
		< 2 (*n* = 297)	≥ 2 (*n* = 1325)		< 2 (*n* = 1054)	≥ 2 (*n* = 568)		< 8 (*n* = 702)	≥ 8 (*n* = 920)		< 9 (*n* = 713)	≥ 9 (*n* = 909)	
In-hospital death	457 (28.2)	66 (22.2)	391 (29.5)	7.3 (2.0–12.6)	250 (23.7)	207 (36.4)	12.7 (8.0–26.3)	146 (20.8)	311 (33.8)	13.0 (8.7–17.3)	145 (20.3)	312 (34.3)	14.0 (9.7–18.3)
	All patients, no (%)	< 2 (*n* = 297)	≥ 2 (*n* = 1325)	Absolute difference, % (95%CI)	< 2 (*n* = 1054)	≥ 2 (*n* = 568)	Absolute difference, % (95%CI)	< 8 (*n* = 702)	≥ 8 (*n* = 920)	Absolute difference, % (95%CI)	< 10 (*n* = 935)	≥ 10 (*n* = 687)	Absolute difference, % (95%CI)
Death within 7 days	280 (17.3)	32 (10.8)	248 (18.7)	8.0 (3.8–12.1)	142 (13.5)	138 (24.3)	10.8 (6.7–14.9)	84 (12.0)	196 (21.3)	9.3 (5.8–12.9)	119 (12.7)	161 (23.4)	12.7 (10.7–15.0)

*Abbreviations: CI* confidence interval, *SIRS* systemic inflammatory response score, *qSOFA* quick sequential organ failure assessment score, *NEWS* national early warning score, *REMS* rapid emergency medicine scores

## Discussion

We validated REMS, SIRS, qSOFA, and NEWS as EWSs for patients with suspected sepsis in the ED. We found that REMS and NEWS performed better than qSOFA and SIRS in predicting adverse outcomes of suspected sepsis patients in the ED. REMS has never been validated for this purpose, and it had the highest discrimination and the highest clinical utility assessed by NB among all the studied EWSs and was well-calibrated.

Identifying patients with life-threatening infection early in the ED is very important. Earlier recognition can lead to earlier initiation of effective and appropriate management. Despite being developed as a component of sepsis definition, SIRS was the first scoring system that was adapted for this purpose. However, it has been criticized for its low specificity [8, 9]. Similarly in our study, SIRS could yield the highest sensitivity, but its sensitivity might have been too high and its specificity not high enough. Over 80% of all patients with suspected sepsis had a SIRS criteria  2, but only 29.5% of them had adverse outcomes while its AUROC showed it to be a poor classifier, which may be worse than a random classifier. Furthermore, SIRS could not demonstrate a higher NB over the treat-all strategy for the in-hospital mortality outcome similar to a previous study [26] and had the lowest NB for the mortality within 7 days of admission outcome. qSOFA was invented and implemented in the Society of Critical Care Medicine and the European Society of Intensive Care Medicine task force 2016 to prompt physicians to suspect sepsis outside the ICU because it could more accurately predict adverse outcomes than SIRS. However, qSOFA has shown to have extremely high specificity at a cost of low sensitivity, especially in the ED where sepsis suspicion usually begins. Our results were also concordant with previous studies [26–28] with qSOFA having a sensitivity as low as < 50%. This could be explained by its low ability to detect sepsis at early disease course. It also could not demonstrate any additional NB on decision-making to a treat-all strategy for in-hospital mortality. Thus, qSOFA may not be an appropriate tool to detect early sepsis at ED presentation.

Our study found that general EWSs performed better than scores developed for sepsis in predicting adverse outcomes associated with sepsis. This could have been because these EWSs have weighted score points and incorporate more physiological components than SIRS and qSOFA. NEWS is a widely used to identify clinically-deteriorating patients, and it has been proven to have better accuracy both in wards and in the ED [16–19]. Similar to previous studies [16, 17], we found that NEWS without baseline risk model could outperform SIRS and qSOFA in AUROC. It also showed higher NB than qSOFA and SIRS over a narrow range of threshold probability.

Interestingly, the EWS with the best accuracy to predict adverse outcome in our study was REMS, which was originally developed to predict mortality in general ED patient with non-specific non-surgical conditions. It has never been validated to predict mortality outcomes after sepsis suspicion in the ED. Our study is the first study to validate REMS for this purpose. We found that REMS performed the best over a wide range of statistical analytic methods. The AUROCs of REMS alone and REMS with baseline risk model was the highest among all the EWSs. The score values were well-calibrated and associated with mortality outcomes. Moreover, it is the EWS with the highest NB over the widest range of threshold probabilities and highest number of avoidable interventions per 100 patients at any particular threshold probability of interest. The superiority of REMS over other EWSs might have been because age is a component of REMS. Older patients might have had higher risk of death secondary to sepsis. Subgroup analyses of patients older than 70 years also showed that all EWSs had better diagnostic and discrimination capacity for both mortality outcomes than in younger patients. REMS was also the best EWS in both subgroups of patients with and without comorbidities. However, we found an opposite trend of AUROCs between the two mortality outcomes in the subgroup analyses comparing patients with at least one and those without comorbidities. This inconsistency might have been because of a small number of participants in the subgroup without comorbidities. In fact, this interpretation should be treated with caution as some of the subgroups contained < 100 outcome events, which might have been too few for an external validation study.

At the recommended cut-point or the best cut-point according to the Youden index, no early warning scores has both high sensitivity and specificity. However, given their wide score ranges, our exploratory analyses of all cut-points of each scoring system revealed that both good rule-in and rule-out properties could be achieved with 2-point cut-offs. With one lower cut-point, more patients could be discharged with lower false negative rate and with another higher cut-point, more patients could be accurately diagnosed with a lower false positive rate. Although these more complex EWSs may be inconvenient for clinical use as a triage tool compared to more easily calculated SIRS and qSOFA, it could be feasible with the increasing use of electronic medical record that could provide automated score calculations as part of the triage process. Nonetheless, it is important to note that the overall prognostic accuracies of all scores were not high enough to be used regardless of clinical signs and symptoms. One should always use them in conjunction with clinical correlation.

Another issue with validating the performance of EWSs for sepsis is the issue of different outcome definitions and how they affect results and interpretations. In-hospital and 30-day mortality were commonly used to provide sufficient numbers of observations for analyses. However, they may depend on health care service provisions and societal preferences/resources for patients and thus not truly represent mortality associated with sepsis. Patients in middle-income countries, unlike high-income countries with hospice facilities and home care, may need to receive their end-of-life care in a hospital. This could be evidenced by the much lower validation AUROCs in our study compared to other studies from higher-income countries, which shows poor transportability of their findings to our setting [16–20]. Moreover, the length of hospital stay range was as much as 310 days and the median time to mortality in our cohort was 27 days (data not shown), indicating prolonged hospital length of stay that may have been due to the lack of hospice service provision in Thailand. Therefore, our mortality within 7 days of admission outcome may be a more valid outcome definition for sepsis-related mortality in our setting and other similar settings. Also, it might be the most generalizable outcome definition to dissimilar settings. NEWS scores were better associated with 7-day mortality than in-hospital mortality. Also, REMS had a NB performance that makes it a more suitable EWS for decision-making with NWT as low as 25 and overall superiority over a treat-all strategy. If using the in-hospital mortality outcome, there is little clinical utility except for a narrow group of patients in terms of harm/cost-to-benefit ratio.

### Limitations

There were several limitations to this study. First, it was conducted in a single tertiary care center in a middle-income country, which may limit the generalizability of the study. Second, we did not have a standardized criterion for sepsis suspicion. Physicians usually based their decisions on patients’ clinical picture, combined with either qSOFA or SIRS. Moreover, we only included patients suspected of sepsis in the ED and not patients that we misdiagnosed and later went on to be diagnosed as sepsis after admission. Including those patients and defining a clearer sepsis suspicion criterion may better represent the true prognostic value of early warning scores. Another limitation is that we only measured one EWS value closest to the time of sepsis suspicion. Using the highest score from repeated measures may improve the accuracy of scoring systems but may not have represented the real clinical situation in the ED, where treatment decisions usually begin at early ED arrival.

## Conclusion

REMS was an early warning score with higher accuracy than sepsis-related scores (qSOFA and SIRS), similar to NEWS. It also had the highest utility in terms of net benefit compared to SIRS, qSOFA and NEWS in predicting in-hospital mortality in patients presenting to the ED with suspected sepsis.

## Supplementary Information

**Additional file 1: Table S1** Components and scores of the SIRS, qSOFA, NEWS and REMS.

**Additional file 2: Table S2** Subgroup analyses of discrimination for in-hospital mortality and mortality within 7 days of admission.

**Additional file 3: Table S3** Pairwise comparisons of area under the receiver operator characteristic curve of baseline mortality risk models and baseline mortality risk models plus early warning scores for in-hospital mortality and mortality within 7 days of admission among patients with suspected sepsis.

**Additional file 4: Table S4** Additional predictive performance contributions of early warning scores to a baseline risk model with gender, age and Charlson Comorbidity Index for all-cause in-hospital mortality and all-cause mortality within 7 days of admission in patients with suspected sepsis.

**Additional file 5: Table S5** Clinical utility of early warning scores for in-hospital mortality and mortality within 7 days of admission among subgroups.

**Additional file 6: Figure S1** Mortality within 7 days of admission stratified by each early warning score in patients with suspected sepsis.

**Additional file 7: Figure S2** Calibration plots of early warning scores for mortality within 7 days in patients with suspected sepsis.

**Additional file 8: Figure S3** Receiver operator characteristic curves for baseline mortality risk model + early warning scores for in-hospital mortality and mortality within 7 days of admission in patients with suspected sepsis.

**Additional file 9: Figure S4** Calibration plots of baseline risk model plus early warning scores for in-hospital mortality in patients with suspected sepsis.

**Additional file 10: Figure S5** Calibration plots of baseline risk model plus early warning scores for mortality within 7 days of admission in patients with suspected sepsis.

**Additional file 11: Figure S6** Decision curves comparing the net benefit of a baseline risk model with gender, age, and Charlson Comorbidity Index + one early warning score for each of SIRS criteria, qSOFA, NEWS and REMS for patients with suspected sepsis at risk of in-hospital mortality and mortality within 7 days of admission over a plausible range of threshold probabilities.

## Data Availability

The dataset is not available but can be requested from the corresponding author.

## Abbreviations

ED: Emergency Department
EWS: Early warning score
SIRS: Systemic Inflammatory Response Syndrome criteria
qSOFA: Quick Sequential Organ Failure Assessment
NEWS: National Early Warning Score
ICU: Intensive care unit
REMS: Rapid Emergency Medicine Score
AUROC: Area under the curve of the receiver operator characteristics curves
NB: Net benefit
NWT: Number willing to treat
LR +: Positive likelihood ratio
LR-: Negative likelihood ration
NPV: Negative predictive value
PPV: Positive predictive value
