# Supplementary material for: The utility of the rapid emergency medicine score (REMS) compared with SIRS, qSOFA and NEWS for Predicting in-hospital Mortality among Patients with suspicion of Sepsis in an emergency department
Source: BMC Emerg Med. 2021 Jan 7;21:2. doi: 10.1186/s12873-020-00396-x (PMC7792356; doi:10.1186/s12873-020-00396-x)
Supplement: Supplementary file 1 — Additional file 1: Table S1 Components and scores of the SIRS, qSOFA, NEWS and REMS. [file 12873_2020_396_MOESM1_ESM.pdf]

Table S1. Components and scores of the SIRS, qSOFA, NEWS and REMS.

**SIRS (0-4)**	**qSOFA (0-3)**	**NEWS (0-20)**	**REMS (0-26)**
HR > 90 /min (1)	Altered mentation (1)	HR (0-3)<br>51-90/min (0), 41-50 or 90-110/min (1), 111-130/min (2), ≤40 or >131/min (3)	HR (0-4)<br>70-109/min (0), 55-69/min or 110-139/min (2), 40-54/min or 140-179/min (3), ≤ 39/min or ≥ 179/min (4)
RR > 20/min or $PaCO_2$<32 mmHg. (1)	RR≥ 22/min (1)	RR (0-3)<br>12-20/min (0), 9-11/min (1), ≤8 or 21-24/min (2), >25/min (3)	RR (0-4)<br>12-24/min (0), 10-11/min or 25-34/min (1), 6-9/min (2), 35-49/min (3), ≤ 5/min or >49/min (4)
Body temperature > 38°c or < 36°c (1)	SBP≤100 mmHg (1)	Body temperature (0-3)<br>36.1-38°c (0), 35.1-36 or 38.1-39°c (1), ≥39.1°c (2), ≤35°c (3)	Age (0-6)<br>< 45 years (0), 45-54 years (2), 55-64 years (3), 65-74 years (5), > 74 years (6)
WBC > 12,000/mm^3 or < 4,000/mm^3, or > 10% band form (1)		SBP (0-3)<br>111-219 mmHg (0), 101-110 mmHg (1), 91-100 mmHg (2) ≤90 or ≥220 mmHg (3)	MAP (0-4)<br>70-109 mmHg. (0), 50-69 mmHg or 110-129 mmHg. (2), 130-159 mmHg. (3), ≤ 49 mmHg. or >159 mmHg. (4)
		Neurological (0-3)<br>Alert (0), reacting to voice or reacting to pain or unresponsive (3)	Glasgow coma score (0-4)<br>14 or 15 (0), 11-13 (1), 8-10 (2), 5-7 (3), 3 or 4 (4)
		Oxygen saturation (0-3)<br>≥96% (0), 94-95% (1), 92-93% (2), ≤91% (3)	Oxygen saturation (0-4)<br>>89% (0), 86-89% (1), 75-85% (3), <75% (4)
		Oxygen supplement (0-2)<br>No (0), Yes (2)	

Abbreviations: SIRS, systemic inflammatory response syndrome criteria; qSOFA, quick sequential organ failure assessment score; NEWS, national early warning score; REMS, rapid emergency medicine score; HR, heart rate; RR, respiratory rate; SBP, systolic blood pressure; MAP, mean arterial pressure; WBC, white blood cell
