# Supplementary material for: The utility of the rapid emergency medicine score (REMS) compared with SIRS, qSOFA and NEWS for Predicting in-hospital Mortality among Patients with suspicion of Sepsis in an emergency department
Source: BMC Emerg Med. 2021 Jan 7;21:2. doi: 10.1186/s12873-020-00396-x (PMC7792356; doi:10.1186/s12873-020-00396-x)
Supplement: Supplementary file 2 — Additional file 2: Table S2 Subgroup analyses of discrimination for in-hospital mortality and mortality within 7 days of admission. [file 12873_2020_396_MOESM2_ESM.pdf]

Table S2. Subgroup analyses of discrimination for in-hospital mortality and mortality within 7 days of admission

	Age		Comorbidities	
	Age<70	Age≥70	Without chronic comorbidities	At least 1 chronic comorbidity
	AUROC (95%CI)	AUROC (95%CI)	AUROC (95%CI)	AUROC (95%CI)
**Discrimination for in-hospital mortality**				
SIRS	0.514 (0.463, 0.564)	0.537 (0.502, 0.573)	0.528 (0.459, 0.597)	0.522 (0.490, 0.553)
qSOFA	0.530 (0.485, 0.579)	0.601 (0.567, 0.634)	0.592 (0.525, 0.658)	0.574 (0.544, 0.605)
NEWS	0.583 (0.531, 0.636)	0.618 (0.580, 0.656)	0.630 (0.551, 0.703)	0.603 (0.568, 0.637)
REMS	0.607 (0.556, 0.678)	0.639 (0.602, 0.673)	0.672 (0.597, 0.742)	0.609 (0.576, 0.642)
**Pairwise AUROC comparisons for in-hospital mortality**				
SIRS vs. qSOFA	0.63	**0.009	0.20	*0.02
SIRS vs. NEWS	*0.02	***<0.001	*0.02	***<0.001
SIRS vs. REMS	**0.005	***<0.001	**0.005	***<0.001
qSOFA vs. NEWS	*0.04	0.35	0.32	0.08
qSOFA vs. REMS	**0.006	0.05	*0.04	0.05
NEWS vs. REMS	0.35	0.26	0.28	0.70
**Discrimination for mortality within 7 days of admission**				
SIRS	0.524 (0.464, 0.582)	0.553 (0.513, 0.593)	0.507 (0.427, 0.590)	0.547 (0.511, 0.584)
qSOFA	0.541 (0.485, 0.601)	0.613 (0.572, 0.653)	0.606 (0.520, 0.687)	0.585 (0.549, 0.623)
NEWS	0.612 (0.548, 0.672)	0.631 (0.587, 0.675)	0.595 (0.496, 0.688)	0.632 (0.593, 0.671)
REMS	0.629 (0.569, 0.688)	0.664 (0.622, 0.706)	0.698 (0.608, 0.779)	0.632 (0.592, 0.670)
**Pairwise AUROC comparisons for mortality within 7 days of admission**				
SIRS vs. qSOFA	0.68	*0.04	0.08	0.15
SIRS vs. NEWS	*0.02	**0.003	0.22	***<0.001
SIRS vs. REMS	**0.005	***<0.001	**0.002	***<0.001
qSOFA vs. NEWS	*0.02	0.39	0.81	*0.02
qSOFA vs. REMS	**0.007	*0.02	0.06	*0.03
NEWS vs. REMS	0.57	0.12	*0.02	0.99

Notes: Pairwise comparisons were by bootstrap test. n of mortality outcome/subgroup N for in-hospital mortality: < 70 years old = 152/587; ≥70 years-old = 305/1035; no chronic comorbidities = 71/362; at least one chronic comorbidity = 386/1260 and for mortality within 7 days of admission n/N: < 70 years-old = 91/587; ≥70 years-old = 189/1035; no chronic comorbidities = 44/362; at least one chronic comorbidity = 236/1260.

Abbreviations: AUROC, area under the receiver operator characteristic curve; CI, confidence interval; SIRS, systemic inflammatory response syndrome; qSOFA, quick Sequential Organ Failure Assessment; NEWS, National Early Warning Score; REMS, Rapid Emergency Medicine Score.
