# Supplementary material for: The utility of the rapid emergency medicine score (REMS) compared with SIRS, qSOFA and NEWS for Predicting in-hospital Mortality among Patients with suspicion of Sepsis in an emergency department
Source: BMC Emerg Med. 2021 Jan 7;21:2. doi: 10.1186/s12873-020-00396-x (PMC7792356; doi:10.1186/s12873-020-00396-x)
Supplement: Supplementary file 3 — Additional file 3: Table S3 Pairwise comparisons of area under the receiver operator characteristic curve of baseline mortality risk models and baseline mortality risk models plus early warning scores for in-hospital mortality and mortality within 7 days of admission among patients with suspected sepsis. [file 12873_2020_396_MOESM3_ESM.pdf]

Table S3. Pairwise comparisons of area under the receiver operator characteristic curve of baseline mortality risk models and baseline mortality risk models plus early warning scores for in-hospital mortality and mortality within 7 days of admission among patients with suspected sepsis

		In-hospital mortality				
		**Baseline model**	**SIRS**	**qSOFA**	**NEWS**	**REMS**
**Mortality within 7 days**	**Baseline model**		*0.02	***<0.001	***<0.001	***<0.001
	**SIRS**	**0.002		0.07	**0.002	***<0.001
	**qSOFA**	***<0.001	0.09		0.17	*0.02
	**NEWS**	***<0.001	*0.001	0.09		0.16
	**REMS**	***<0.001	***<0.001	**0.006	0.15	

Notes: comparisons of baseline risk model vs. + EWS were performed by likelihood ratio test for nested models, and other pairwise comparison were performed by bootstrap test.

Abbreviations: NEWS, National Early Warning Score; qSOFA, quick Sequential Organ Failure Assessment; REMS, Rapid Emergency Medicine Score; SIRS, systemic inflammatory response syndrome. Notes:- *p<0.05 **p<0.01 ***p<0.001
