# Supplementary material for: The utility of the rapid emergency medicine score (REMS) compared with SIRS, qSOFA and NEWS for Predicting in-hospital Mortality among Patients with suspicion of Sepsis in an emergency department
Source: BMC Emerg Med. 2021 Jan 7;21:2. doi: 10.1186/s12873-020-00396-x (PMC7792356; doi:10.1186/s12873-020-00396-x)
Supplement: Supplementary file 4 — Additional file 4: Table S4 Additional predictive performance contributions of early warning scores to a baseline risk model with gender, age and Charlson Comorbidity Index for all-cause in-hospital mortality and all-cause mortality within 7 days of admission in patients with suspected sepsis. [file 12873_2020_396_MOESM4_ESM.pdf]

Table S4. Additional predictive performance contributions of early warning scores to a baseline risk model with gender, age and Charlson Comorbidity Index for all-cause in-hospital mortality and all-cause mortality within 7 days of admission in patients with suspected sepsis

	**Discrimination**	**Discrimination improvement**			**Calibration**	**Overall performance**	
	**AUROC (95%CI)**	**IDI Baseline + EWS vs. Baseline (95%CI)**	**Change in sensitivity (%)**	**Change in specificity (%)**	**Hosmer-Lemeshow test**	**Scaled Brier score (%)**	**Nagelkerke's R square (%)**
			**In-hospital mortality**				
Baseline model	0.576 (0.547, 0.608)	-	-	-	0.211	1.3	1.3
Baseline + SIRS	0.584 (0.553, 0.614)	0.003 (0.000, 0.011)	0.795	- 0.327	0.185	1.6	1.6
Baseline + qSOFA	0.609 (0.577, 0.639)	0.020 (0.009, 0.038)	4.95	- 2.04	0.150	3.3	3.2
Baseline + NEWS	0.626 (0.596, 0.657)	0.029 (0.014, 0.048)	7.04	- 2.89	0.143	4.2	4.0
Baseline + REMS	0.643 (0.613, 0.673)	0.040 (0.024, 0.062)	9.98	- 4.10	0.318	5.4	5.2
			**Mortality within 7 days of admission**				
Baseline model	0.612 (0.577, 0.647)	-	-	-	0.128	2.2	2.2
Baseline + SIRS	0.621 (0.586, 0.656)	0.006 (0.001, 0.016)	2.71	- 0.641	0.421	2.8	2.7
Baseline + qSOFA	0.647 (0.610, 0.682)	0.018 (0.006, 0.035)	7.64	- 1.80	0.276	3.9	3.8
Baseline + NEWS	0.670 (0.577, 0.647)	0.032 (0.017, 0.054)	14.0	- 3.31	0.340	5.1	5.0
Baseline + REMS	0.688 (0.655, 0.721)	0.042 (0.025, 0.068)	18.3	- 4.32	0.075	6.2	6.1

Notes: change in sensitivity was calculated as $\left(average\ \widehat{Pr}\ events\ baseline + EWS\ model -\ average\ \widehat{Pr}\ events\ baseline\ model\right) \div\ average\ \widehat{Pr}\ eventsbaseline\ model$. Change in specificity was calculated as $\left(average\ \widehat{Pr}\ nonevents\ baseline + EWS\ model -\ average\ \widehat{Pr}\ nonevents\ baseline\ model\right) \div\ average\ \widehat{Pr}\ nonevents\ baseline\ model$. Abbreviations= AUROC, area under the receiver operator characteristic curve; CI, confidence interval; IDI, integrated discrimination index; EWS, early warning score; SIRS, systemic inflammatory response syndrome; qSOFA, quick Sequential Organ Failure Assessment; NEWS, National Early Warning Score; REMS, Rapid Emergency Medicine Score.
