# Supplementary material for: The utility of the rapid emergency medicine score (REMS) compared with SIRS, qSOFA and NEWS for Predicting in-hospital Mortality among Patients with suspicion of Sepsis in an emergency department
Source: BMC Emerg Med. 2021 Jan 7;21:2. doi: 10.1186/s12873-020-00396-x (PMC7792356; doi:10.1186/s12873-020-00396-x)
Supplement: Supplementary file 5 — Additional file 5: Table S5 Clinical utility of early warning scores for in-hospital mortality and mortality within 7 days of admission among subgroups. [file 12873_2020_396_MOESM5_ESM.pdf]

Table S5. Clinical utility of early warning scores for in-hospital mortality and mortality within 7 days of admission among subgroups

	**Sensitivity (95%CI)**	**Specificity (95%CI)**	**PPV (95%CI)**	**NPV (95%CI)**	**LR+ (95%CI)**	**LR- (95%CI)**
	**In-hospital mortality**					
	**Age < 70**					
SIR ≥ 2	85.5 (78.9, 90.7)	14.0 (10.9, 17.7)	25.9 (24.4, 27.3)	73.5 (63.9, 81.3)	1.0 (0.9, 1.1)	1.0 (0.7, 1.6)
qSOFA ≥ 2	39.5 (31.7, 47.7)	68.1 (63.4, 72.4)	30.2 (25.4, 35.4)	76.3 (73.6, 78,8)	1.2 (1.0, 1.6)	0.9 (0.8, 1.0)
NEWS ≥ 8	69.1 (61.1, 76.3)	44.6 (39.9, 49.4)	30.4 (27.6, 33.3)	80.5 (76.1, 84.3)	1.3 (1.1, 1.4)	0.7 (0.5, 0.9)
REMS ≥ 9	44.1 (36.0, 52.4)	70.8 (66.3, 75.0)	34.5 (29.5, 39.9)	78.4 (75.7, 80.9)	1.5 (1.2, 1.9)	0.8 (0.7, 0.9)
	**Age ≥ 70**					
SIR ≥ 2	85.6 (81.1, 89.3)	23.4 (20.3, 26.5)	31.8 (30.5, 33.1)	79.4 (74.0, 84.0)	1.1 (1.1, 1.2)	0.6 (0.5, 0.8)
qSOFA ≥ 2	48.2 (42.5, 54.0)	69.6 (66.1, 72.9)	39.8 (36.1, 43.7)	76.3 (74.1, 78.4)	1.6 (1.4, 1.9)	0.7 (0.7, 0.8)
NEWS ≥ 8	67.5 (62.0, 72.8)	49.6 (45.9, 53.3)	35.9 (33.5, 38.4)	78.5 (75.4, 81.4)	1.3 (1.2, 1.5)	0.7 (0.6, 0.8)
REMS ≥ 9	80.3 (75.4, 84.6)	35.6 (32.1, 39.2)	34.3 (32.5, 36.0)	81.3 (77.2, 84.7)	1.3 (1.2, 1.4)	0.6 (0.4, 0.7)
	**Without chronic comorbidities**					
SIR ≥ 2	83.1 (72.3, 91.0)	18.8 (14.8, 23.3)	20.0 (18.2, 21.9)	82.0 (72.2, 88.9)	1.0 (0.9, 1.1)	0.9 (0.5, 1.6)
qSOFA ≥ 2	47.9 (35.9, 60.1)	70.1 (64.5, 75.3)	28.1 (22.5, 34.5)	84.7 (81.3, 87.5)	1.6 (1.2, 2.2)	0.7 (0.6, 0.9)
NEWS ≥ 8	71.8 (60.0, 81.9)	46.1 (40.2, 52.0)	24.5 (21.3, 28.0)	87.0 (81.9, 90.8)	1.3 (1.1, 1.6)	0.6 (0.4, 0.9)
REMS ≥ 9	70.4 (58.4, 80.7)	53.6 (47.7, 59.5)	27.0 (23.4, 31.0)	88.1 (83.6, 91.5)	1.5 (1.2, 1.8)	0.6 (0.4, 0.8)
	**At least 1 chronic comorbidity**					
SIR ≥ 2	86.0 (82.1, 89.3)	19.1 (16.6, 21.9)	32.0 (30.8, 33.1)	75.6 (70.0, 80.4)	1.1 (1.0, 1.1)	0.7 (0.6, 1.0)
qSOFA ≥ 2	44.8	68.7	38.7	73.8	1.4	0.8

	(40.0, 49.9)	(65.5, 71.7)	(35.3, 42,3)	(71.8, 75.7)	(1.2, 1.7)	(0.7, 0.9)
NEWS ≥ 8	67.4<br>(62.4, 72.0)	48.3<br>(44.9, 51.7)	36.5<br>(34.4, 38.7)	77.0<br>(74.1, 79.7)	1.3<br>(1.2, 1.4)	0.7<br>(0.6, 0.8)
REMS ≥ 9	67.9<br>(63.0, 72.5)	47.1<br>(43.8, 50.5)	36.2<br>(34.1, 38.4)	76.9<br>(73.9, 79.6)	1.3<br>(1.2, 1.4)	0.7<br>(0.6, 0.8)
	**Mortality within 7 days of admission**					
	**Age < 70**					
SIR ≥ 2	90.1<br>(82.1, 95.4)	14.9<br>(11.9, 18.4)	16.3<br>(15.2, 17.4)	89.2<br>(81.0, 94.1)	1.1<br>(0.9, 1.1)	0.7<br>(0.3, 1.3)
qSOFA ≥ 2	42.9<br>(32.5, 53.7)	67.7<br>(63.4, 71.8)	19.6<br>(15.7, 24.2)	86.6<br>(84.3, 88.6)	1.3<br>(1.0, 1.7)	0.8<br>(0.7, 1.0)
NEWS ≥ 8	71.4<br>(61.0, 80.4)	43.4<br>(38.9, 47.8)	18.8<br>(16.6, 21.2)	89.2<br>(85.5, 92.1)	1.3<br>(1.1, 1.5)	0.7<br>(0.5, 0.9)
REMS ≥ 10	36.3<br>(26.4, 47.0)	77.0<br>(73.1, 80.7)	22.5<br>(17.4, 28.4)	86.8<br>(84.9, 88.6)	1.6<br>(1.2, 2.2)	0.8<br>(0.7, 1.0)
	**Age ≥ 70**					
SIR ≥ 2	87.8<br>(82.3, 92.1)	22.6<br>(19.8, 25.6)	20.2<br>(19.2, 21.3)	89.3<br>(84.7, 92.6)	1.1<br>(1.1, 1.2)	0.5<br>(0.4, 0.8)
qSOFA ≥ 2	52.4<br>(45.0, 59.7)	68.1<br>(64.8, 71.2)	26.8<br>(23.7, 30.3)	86.5<br>(84.6, 88.2)	1.6<br>(1.4, 1.9)	0.7<br>(0.6, 0.8)
NEWS ≥ 8	69.3<br>(62.2, 75.8)	47.6<br>(44.2, 51.1)	22.8<br>(20.9, 24.9)	87.4<br>(84.7, 89.7)	1.3<br>(1.2, 1.5)	0.6<br>(0.5, 0.8)
REMS ≥ 10	67.7<br>(60.6, 74.3)	51.3<br>(47.8, 54.7)	23.7<br>(21.6, 26.0)	87.7<br>(85.1, 89.8)	1.4<br>(1.2, 1.6)	0.6<br>(0.5, 0.8)
	**Without chronic comorbidities**					
SIR ≥ 2	81.8<br>(67.3, 91.8)	21.4<br>(17.0, 26.3)	12.6<br>(11.0, 14.3)	89.5<br>(81.5, 94.3)	1.0<br>(0.9, 1.2)	0.9<br>(0.4, 1.7)
qSOFA ≥ 2	52.3<br>(36.7, 67.5)	69.2<br>(65.8, 74.2)	19.0<br>(14.5, 24.5)	91.3<br>(88.4, 93.5)	1.7<br>(1.2, 2.4)	0.7<br>(0.5, 1.0)
NEWS ≥ 8	65.9<br>(50.1, 79.5)	43.7<br>(38.2, 49.4)	13.9<br>(11.4, 17.0)	90.3<br>(85.8, 93.4)	1.2<br>(0.9, 1.5)	0.8<br>(0.5, 1.2)
REMS ≥ 10	61.4<br>(45.5, 75.6)	63.8<br>(58.3, 69.1)	19.0<br>(15.1, 23.6)	92.3<br>(89.1, 94.6)	1.7<br>(1.3, 2.2)	0.6<br>(0.4, 0.9)
	**At least 1 chronic comorbidity**					
SIR ≥ 2	89.8<br>(85.3, 93.4)	19.2<br>(16.9, 21.8)	20.4<br>(19.6, 21.3)	89.1<br>(84.6, 92.5)	1.1<br>(1.1, 1.2)	0.5<br>(0.4, 0.8)

qSOFA > 2	48.7<br>(42.2, 55.3)	67.6<br>(64.6, 70.4)	25.7<br>(22.8, 28.9)	85.1<br>(83.4, 86.7)	1.5<br>(1.3, 1.8)	0.8<br>(0.7, 0.9)
NEWS > 8	70.8<br>(64.5, 76.5)	46.8<br>(43.7, 49.9)	23.5<br>(21.7, 25.3)	87.4<br>(84.9, 89.5)	1.3<br>(1.2, 1.5)	0.6<br>(0.5, 0.8)
REMS > 10	56.8<br>(50.2, 63.2)	59.9<br>(56.8, 62.9)	24.6<br>(22.2, 27.2)	85.7<br>(83.7, 87.5)	1.4<br>(1.2, 1.6)	0.7<br>(0.6, 0.8)

Notes: n of mortality outcome/subgroup N for in-hospital mortality: < 70 years-old = 152/587; >70 years-old = 305/1035; no chronic comorbidities = 71/362; at least one chronic comorbidity = 386/1260 and for mortality within 7 days of admission n/N: < 70 years-old = 91/587; >70 years-old = 189/1035; no chronic comorbidities = 44/362; at least one chronic comorbidity = 236/1260

Abbreviations: CI, confidence interval; SIRS, systemic inflammatory response syndrome; qSOFA, quick Sequential Organ Failure Assessment; NEWS, National Early Warning Score; REMS, Rapid Emergency Medicine Score.
