# Supplementary material for: The utility of the rapid emergency medicine score (REMS) compared with SIRS, qSOFA and NEWS for Predicting in-hospital Mortality among Patients with suspicion of Sepsis in an emergency department
Source: BMC Emerg Med. 2021 Jan 7;21:2. doi: 10.1186/s12873-020-00396-x (PMC7792356; doi:10.1186/s12873-020-00396-x)
Supplement: Supplementary file 6 — Additional file 6: Figure S1 Mortality within 7 days of admission stratified by each early warning score in patients with suspected sepsis. [file 12873_2020_396_MOESM6_ESM.pdf]

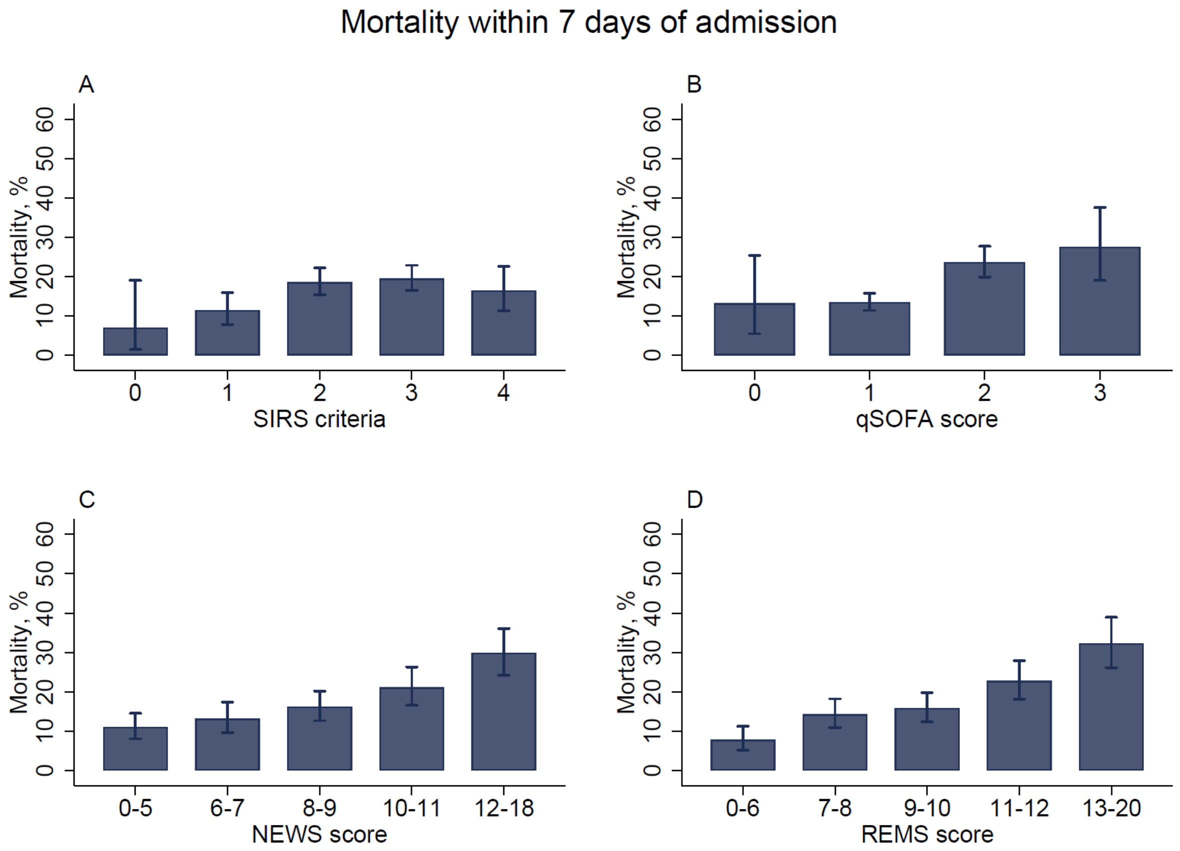

Figure S1. Mortality within 7 days of admission stratified by each early warning score in patients with suspected sepsis.

(A) SIRS criteria. (B) qSOFA score. (C) NEWS score. (D) REMS score. Error bars denote 95% confidence intervals. NEWS and REMS scores were categorized into 5 groups by quintiles of each score.

Abbreviations: SIRS, systemic inflammatory response syndrome; qSOFA, quick Sequential Organ Failure Assessment; NEWS, National Early Warning Score; REMS, Rapid Emergency Medicine Score.
