# Supplementary material for: The utility of the rapid emergency medicine score (REMS) compared with SIRS, qSOFA and NEWS for Predicting in-hospital Mortality among Patients with suspicion of Sepsis in an emergency department
Source: BMC Emerg Med. 2021 Jan 7;21:2. doi: 10.1186/s12873-020-00396-x (PMC7792356; doi:10.1186/s12873-020-00396-x)
Supplement: Supplementary file 8 — Additional file 8: Figure S3 Receiver operator characteristic curves for baseline mortality risk model + early warning scores for in-hospital mortality and mortality within 7 days of admission in patients with suspected sepsis. [file 12873_2020_396_MOESM8_ESM.pdf]

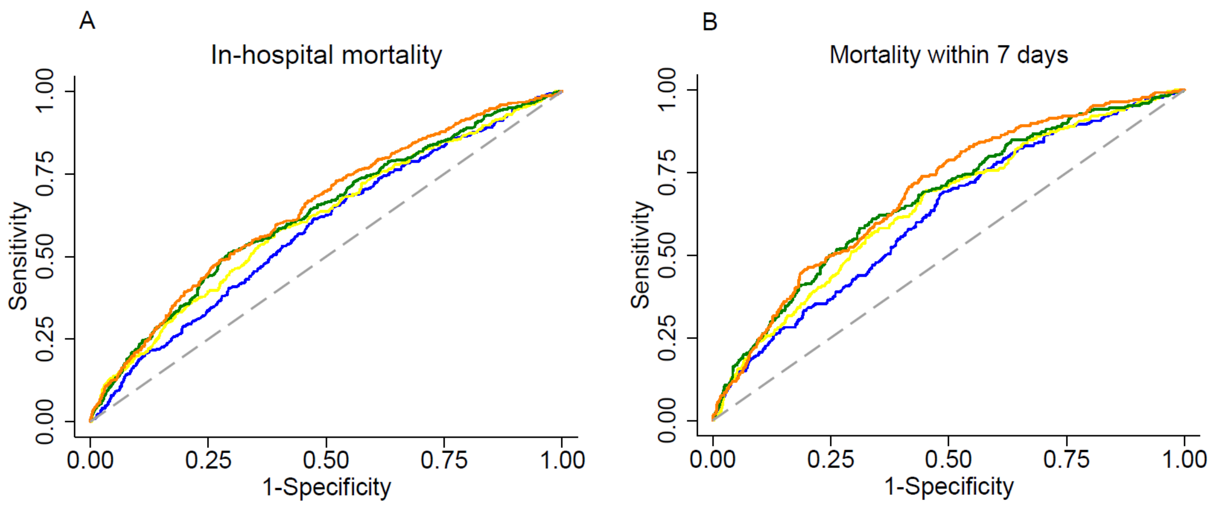

Figure S3. Receiver operator characteristic curves for baseline mortality risk model + early warning scores for in-hospital mortality and mortality within 7 days of admission in patients with suspected sepsis.

(A) In-hospital mortality. (B) Mortality within 7 days of admission. EWS score = SIRS (blue line), qSOFA (yellow line),), NEWS (green line), and REMS (orange line). Abbreviations: SIRS, systemic inflammatory response syndrome; qSOFA, quick Sequential Organ Failure Assessment; NEWS, National Early Warning Score; REMS, Rapid Emergency Medicine Score.
