# Supplementary material for: The utility of the rapid emergency medicine score (REMS) compared with SIRS, qSOFA and NEWS for Predicting in-hospital Mortality among Patients with suspicion of Sepsis in an emergency department
Source: BMC Emerg Med. 2021 Jan 7;21:2. doi: 10.1186/s12873-020-00396-x (PMC7792356; doi:10.1186/s12873-020-00396-x)
Supplement: Supplementary file 9 — Additional file 9: Figure S4 Calibration plots of baseline risk model plus early warning scores for in-hospital mortality in patients with suspected sepsis. [file 12873_2020_396_MOESM9_ESM.pdf]

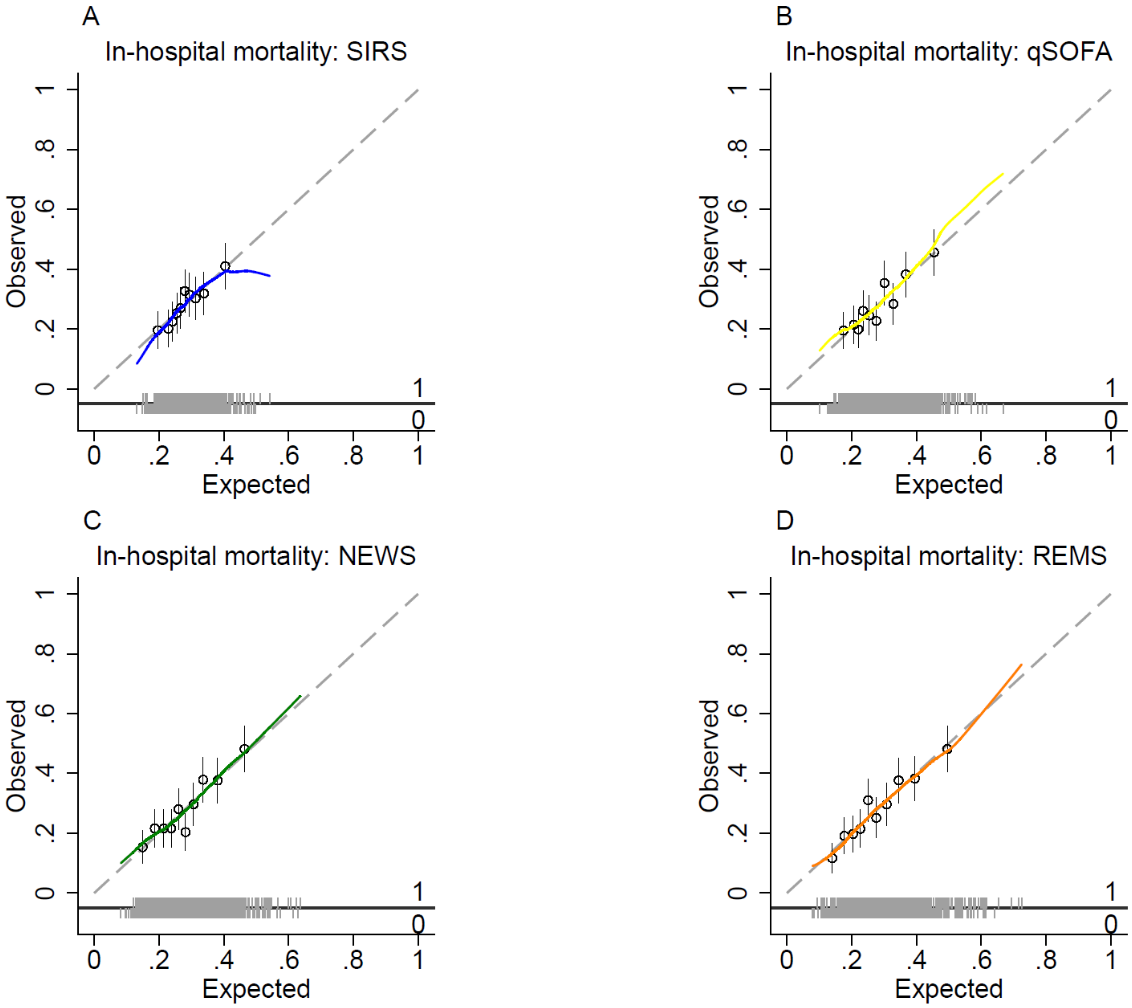

Figure S4. Calibration plots of baseline risk model plus early warning scores for in-hospital mortality in patients with suspected sepsis.

(A) SIRS criteria. (B) qSOFA score. (C) NEWS score. (D) REMS score. Hollow circles denote groups of predicted risk. Vertical line through hollow circles denote 95% confidence intervals. The distribution of non-events of the outcome (0) and events of the outcome (1) by expected probability are denoted by the rug plot (light grey) along the x axis.

Abbreviations: SIRS, systemic inflammatory response syndrome; qSOFA, quick Sequential Organ Failure Assessment; NEWS, National Early Warning Score; REMS, Rapid Emergency Medicine Score.
