# Supplementary material for: The utility of the rapid emergency medicine score (REMS) compared with SIRS, qSOFA and NEWS for Predicting in-hospital Mortality among Patients with suspicion of Sepsis in an emergency department
Source: BMC Emerg Med. 2021 Jan 7;21:2. doi: 10.1186/s12873-020-00396-x (PMC7792356; doi:10.1186/s12873-020-00396-x)
Supplement: Supplementary file 10 — Additional file 10: Figure S5 Calibration plots of baseline risk model plus early warning scores for mortality within 7 days of admission in patients with suspected sepsis. [file 12873_2020_396_MOESM10_ESM.pdf]

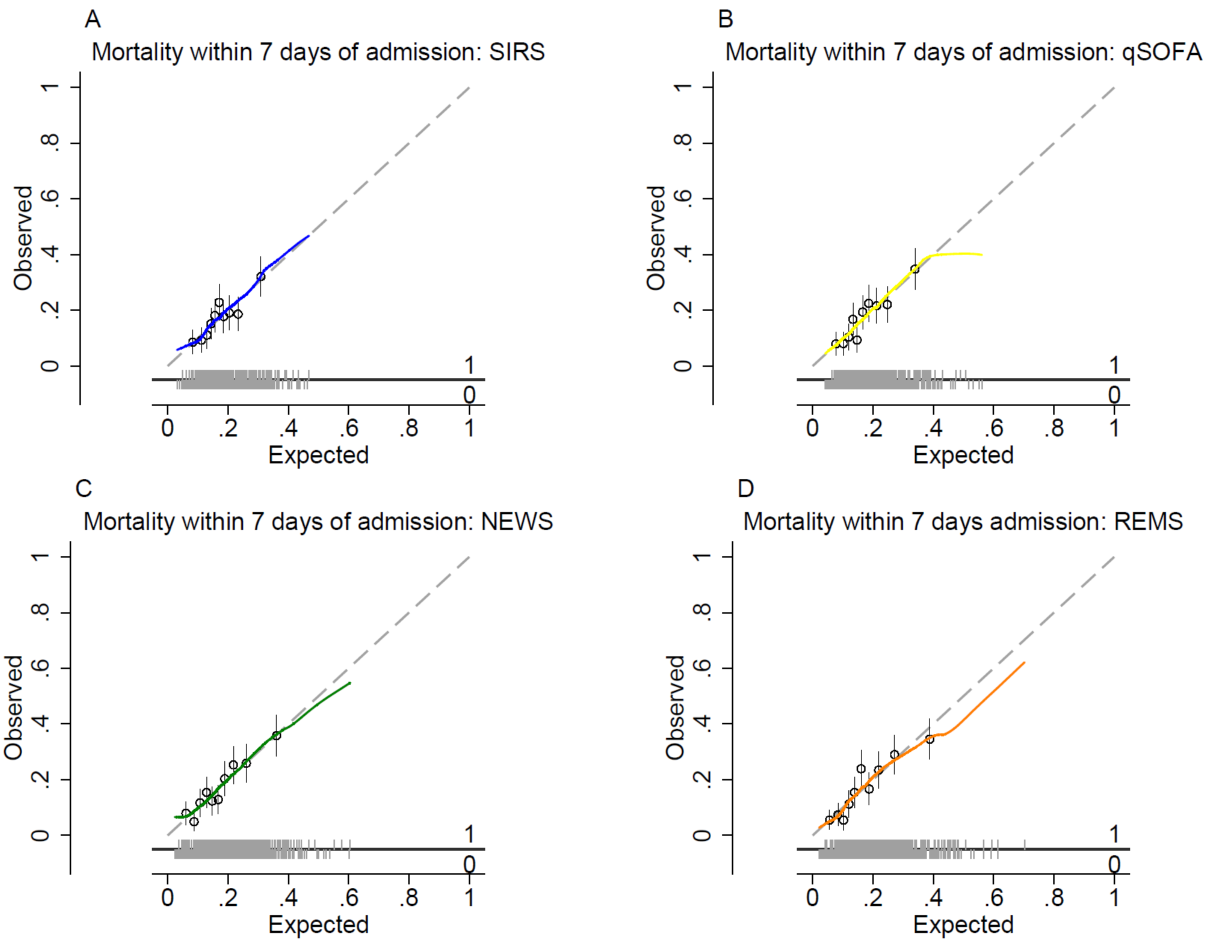

Figure S5. Calibration plots of baseline risk model plus early warning scores for mortality within 7 days of admission in patients with suspected sepsis.

(A) SIRS criteria. (B) qSOFA score. (C) NEWS score. (D) REMS score. Hollow circles denote groups of predicted risk used to fit a smoothed curve. Vertical line through hollow circles denote 95% confidence intervals. The distribution of non-events of the outcome (0) and events of the outcome (1) by expected probability are denoted by the rug plot (light grey) along the x axis.

Abbreviations: SIRS, systemic inflammatory response syndrome; qSOFA, quick Sequential Organ Failure Assessment; NEWS, National Early Warning Score; REMS, Rapid Emergency Medicine Score.
