# Supplementary material for: The utility of the rapid emergency medicine score (REMS) compared with SIRS, qSOFA and NEWS for Predicting in-hospital Mortality among Patients with suspicion of Sepsis in an emergency department
Source: BMC Emerg Med. 2021 Jan 7;21:2. doi: 10.1186/s12873-020-00396-x (PMC7792356; doi:10.1186/s12873-020-00396-x)
Supplement: Supplementary file 11 — Additional file 11: Figure S6 Decision curves comparing the net benefit of a baseline risk model with gender, age, and Charlson Comorbidity Index + one early warning score for each of SIRS criteria, qSOFA, NEWS and REMS for patients with suspected sepsis at risk of in-hospital mortality and mortality within 7 days of admission over a plausible range of threshold probabilities. [file 12873_2020_396_MOESM11_ESM.pdf]

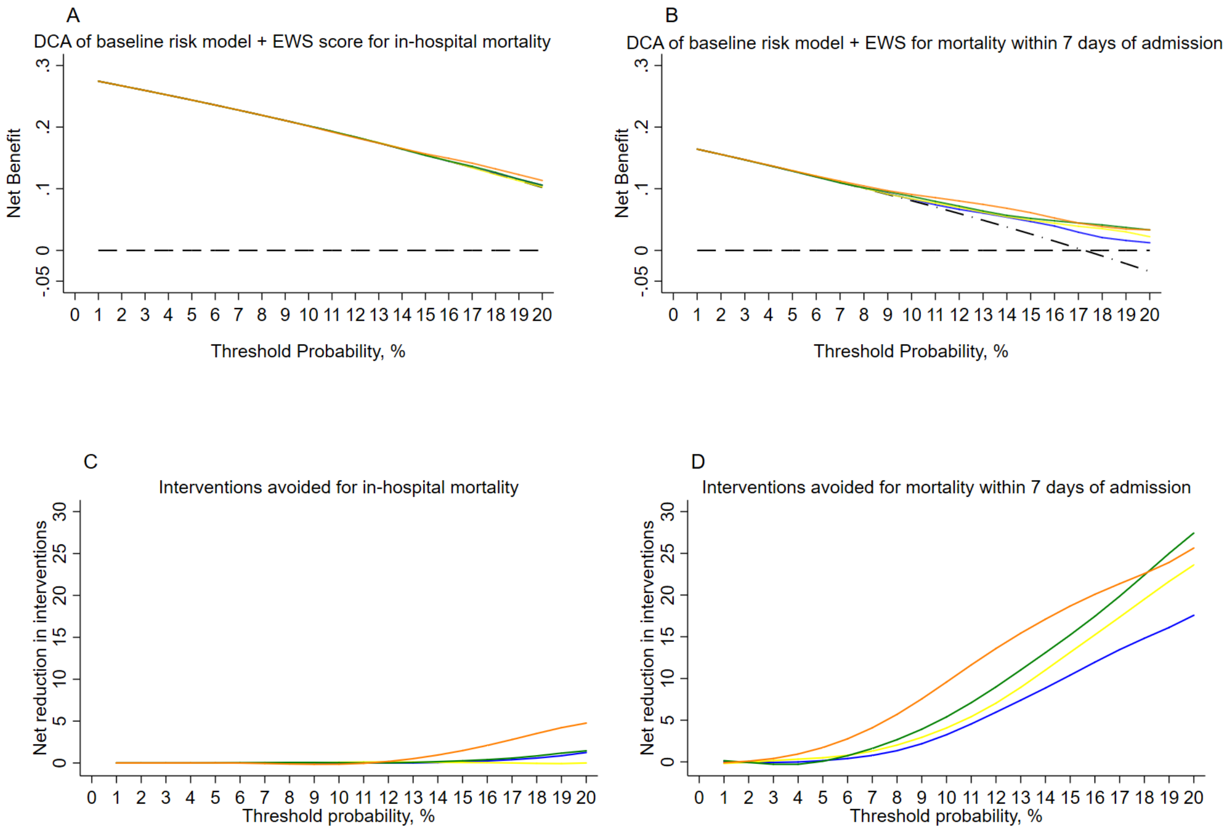

Figure S6. Decision curves comparing the net benefit of a baseline risk model with gender, age, and Charlson Comorbidity Index + one early warning score for each of SIRS criteria, qSOFA, NEWS and REMS for patients with suspected sepsis at risk of (A) in-hospital mortality and (B) mortality within 7 days of admission over a plausible range of threshold probabilities.

SIRS criteria (blue line), qSOFA (yellow line), NEWS (green line), and REMS (orange line), a 'treat all' strategy (black dashed and dotted line), and a 'treat none' strategy (black dashed line) Net benefit = (true-positive classifications – harm/cost-to-benefit ratio × false-positive classifications) ÷ N. The threshold probability represents the number of patients that the physician is willing to treat. Net reduction in interventions per 100 patients in a theoretical population for (C) in-hospital mortality and (D) mortality within 7 days of admission. Abbreviations: SIRS, systemic inflammatory response syndrome; qSOFA, quick Sequential Organ Failure Assessment; NEWS, National Early Warning Score; REMS, Rapid Emergency Medicine Score.
